# *Laf4/Aff3*, a Gene Involved in Intellectual Disability, Is Required for Cellular Migration in the Mouse Cerebral Cortex

**DOI:** 10.1371/journal.pone.0105933

**Published:** 2014-08-27

**Authors:** Justin M. Moore, Peter L. Oliver, Mattéa J. Finelli, Sheena Lee, Tom Lickiss, Zoltán Molnár, Kay E. Davies

**Affiliations:** 1 MRC Functional Genomics Unit, University of Oxford, Oxford, United Kingdom; 2 Department of Physiology, Anatomy and Genetics, University of Oxford, Oxford, United Kingdom; CNRS UMR7275, France

## Abstract

Members of the AFF (AF4/FMR2) family of putative transcription factors are involved in infant acute leukaemia and intellectual disability (ID), although very little is known about their transcriptional targets. For example, deletion of human lymphoid nuclear protein related to AF4*/*AFF member 3 (*LAF4/AFF3)* is known to cause severe neurodevelopmental defects, and silencing of the gene is also associated with ID at the folate-sensitive fragile site (FSFS) FRA2A; yet the normal function of this gene in the nervous system is unclear. The aim of this study was to further investigate the function of *Laf4* in the brain by focusing on its role in the cortex. By manipulating expression levels in organotypic slices, we demonstrate here that *Laf4* is required for normal cellular migration in the developing cortex and have subsequently identified *Mdga2*, an important structural protein in neurodevelopment, as a target of Laf4 transcriptional activity. Furthermore, we show that the migration deficit caused by loss of *Laf4* can be partially rescued by *Mdga2* over-expression, revealing an important functional relationship between these genes. Our study demonstrates the key transcriptional role of *Laf4* during early brain development and reveals a novel function for the gene in the process of cortical cell migration relevant to the haploinsufficiency and silencing observed in human neurodevelopmental disorders.

## Introduction

Mixed lineage leukaemia (*MLL)* gene rearrangements occur in approximately 70 percent of infant acute lymphoblastic leukaemia (ALL) patients [1]. Lymphoid nuclear protein related to AF4 (*LAF4*, also known as *AFF3*) is one of an estimated 40 genes that can form such MLL fusions [2], although *LAF4* is one of the few genes that is aberrantly translocated in both B- and T-cell derived leukemia [3]. Moreover, *LAF4* has been found to be abnormally expressed in approximately 20 percent of breast cancers, suggesting that it may act as a proto-oncogene [4]. A human microdeletion of 500 kb on chromosome 2q11.1 encompassing only the *LAF4* gene has been detected by array comparative genomic hybridization on peripheral lymphocytes [5]. The patient presented with developmental delay, seizures, urogenital and limb defects and died at four months of age after numerous repeated apnoeic episodes [5]. The patient was also noted to be of low birth weight (10^th^ centile), and magnetic resonance imaging (MRI) revealed a dilated ventricular system with cortical and subcortical brain atrophy. In addition, a recent study identified a CGG repeat expansion in the promoter of *LAF4* at an autosomal folate-sensitive fragile site (FSFS) named FRA2A that is associated with ID [6]. It was shown that this polymorphic repeat is hypermethylated in FRA2A leading to silencing of *LAF4* in the nervous system [6]. However, the functional consequences of such a reduction in *LAF4* expression are unknown.

LAF4 is one of four members of the AFF (AF4/FMR2) protein family in higher mammals, consisting of ALL-1 fused gene from chromosome 4 (AF4 or AFF1), Fragile X mental retardation 2 (FMR2 or AFF2) and ALL-fused gene from 5q31 (AF5Q31 or AFF4). Like LAF4, AF4 and AF5Q31 are known to form fusion proteins with MLL [7], [8], whereas FMR2 is silenced in FRAXE (mental retardation, X-linked, associated with fragile site) intellectual disability and is not implicated in ALL [9], [10]. While a mouse *Fmr2* null mutant does show some subtle behavioral and electrophysiological deficits related to synaptic plasticity [11], gene knockouts of *Af4* and *Af5q31* have not revealed a great deal regarding the normal molecular function of AFF proteins [12], [13] and no *Laf4* mutants have been reported to date. Interestingly, a dominant point mutation in *Af4* in the *robotic* ataxic mutant mouse reduces the turn-over of the protein by seven in absentia homolog (SIAH) proteins, a family of E3 ubiquitin-protein ligases; these data have revealed the importance of Af4 in the survival of Purkinje neurons in the cerebellum [14], [15].

AFF proteins were originally described as putative transcription factors based on the presence of a conserved transactivation domain [16]. Importantly, subsequent biochemical studies demonstrated the key role that AFF proteins play in mediating transcriptional activity, revealing an association with the positive transcription elongation factor b (P-TEFb) [17]. P-TEFb, AF4 and ENL/AF9 form a large complex capable of interacting with RNA polymerase II (Pol II) and this complex can also interact with disruptor of telomeric silencing (DOT1) to enhance methylation at histone 3 lysine residue 79 (H3K79) [17]. This complex has since become widely studied as a source of aberrant transcriptional activity related to oncogenesis in MLL/AFF protein fusion events [18], [19] and more recently in the direct transcriptional control of integrated HIV genomes [20]. Further *in* *vitro* data have shown that all members of the AFF family form nuclear foci, also termed nuclear speckles, which may be important in regulating splicing events through interactions with pre-mRNA factors [15], [21].

Despite these studies, very little is known about the normal transcriptional targets of AFF proteins, as only very few have been confirmed [22], [23]. Furthermore, in view of the relevance of *FMR2* and *LAF4* to neurodevelopmental disease and cerebellar neurodegeneration in the *Af4* mutant mouse *robotic*, the role of AFF proteins in the CNS warrants further investigation. For example, specific temporal patterns of *Fmr2* and *Laf4* expression have been described in the developing mouse embryo; whereas *Fmr2* expression peaks at late embryonic stages [11], *Laf4* was shown to be expressed in the developing cortex as early as embryonic day (E)13.5 [24]. At E13.5, *Laf4* is also detected in cartilage tissue in different regions of the embryo as well as in the lung, kidney tubules and bladder [24]. A similar pattern has been confirmed in humans, with very high levels of *LAF4* seen in the fetal brain, which then diminished to much lower levels in adults [25].

In light of the reported human *LAF4* deletion, association with ID and known expression patterns, the aim of this study was to further investigate the function of Laf4 in the brain, focusing on the role of the protein in the developing cortex. By manipulating expression levels of *Laf4* by whole embryo electroporation followed by organotypic culture experiments, we discovered that *Laf4* is required for cortical cell migration. We then went on to examine the potential targets of Laf4 transcriptional control and found that Laf4 regulates the expression of Mdga2, an important structural protein in the developing CNS. These data represent the first detailed functional studies of Laf4 and highlight the importance of the gene in the developing nervous system with relevance to human neurodevelopmental disorders.

## Materials and Methods

### Cloning

A full-length cDNA representing mouse *Laf4* (corresponding to NCBI accession number NM_010678.2) with an additional HA-tag at the C-terminus was cloned into the IRES-GFP vector (a gift from Dr Edward Turner [26]) to generate Laf4-HA-IRES-GFP. For *Laf4* knockdown, complementary shRNA oligonucleotides were cloned into the LentiLox 3.7 (pLL.3) vector (Science Gateway) using the following primers: 5′-TGGACCTTGCTTGGAGATG GTGGTTCAAGAGAACACCATCTCCAAGCAAGGTCCTTTTTC-3′, 5′-TCGAGAAAAAGGACCTTGCTTGGAGATGGTGTTCTCTTGAACCACC ATCTCCAAGCAAGGTCCA-3′. Site directed mutagenesis was used to introduce three silent single nucleotide mutations into Laf4-HA-IRES-GFP at the shRNA-Laf4 target sequence to generate Laf4-HA-rescue-IRES-GFP (for shRNA primer sequences see Table S1). The scramble construct was designed to match the G:C/A:T ratio of the shRNA-Laf4 knockdown construct and to avoid predicted sequence alignment with the mouse genome. A full-length cDNA representing mouse *Mdga2* (NM_001193266) was cloned into the same vector to generate Mdga2-IRES-GFP. The additional shRNAs tested were obtained from Sigma (Mission shRNAs to XM_283603, TRCN0000086608-12).

### *In situ* hybridization

A region of the *Laf4* coding sequence corresponding to a ∼300 bp sequence at the 3′ end of the mRNA transcript was amplified by RT-PCR using primers 5′-GATGAACTCTCCAACCGGATC and 5′-AGCCCATGGCACCTCTCTTG. The PCR product was cloned into pCR4-TOPO (Invitrogen) for DIG-labelled riboprobe synthesis from linearised plasmid DNA. Probes were hybridized to 14 µM frozen sections as previously described [14].

### Animals

All animal experiments have been approved by the UK Home Office and the University of Oxford Ethical Review Panel.

### Cortical cell culture and gene electroporation

E14.5 embryos or P1 neonates were dissected on ice and cortical cells were dissociated and electroporated as per the manufacturer’s protocol. Briefly, after trypsin digestion, trituration and washes, cells (2.7×10^5^/cm^2^) were mixed with Nucleofector solution (Amaxa), plasmid DNA and electroporated as recommended by the manufacturer. Cells were re-suspended in culture medium (Neurobasal, 5% horse serum, 2% B27 supplement and 2 mM Glutamax (Gibco, UK)) and plated on wells pre-coated with poly-L-lysine. Cells were then cultured for 96 hours or 60 hours, with half the medium being changed every two days.

### Western blotting

Total protein extracts from cells were prepared and incubated on ice for 45 minutes in lysis buffer (150 mM NaCl, 50 mM Tris-HCl pH 7.0, 1% Triton X-100, 0.1% SDS) containing protease inhibitors (Sigma, UK). Following centrifugation to clarify lysates, protein levels were then quantified using the BCA assay kit (Pierce, UK). Proteins were run on a 10% SDS-PAGE gel and transferred onto a PVDF membrane (Amersham Biosciences, UK). Membranes were blocked in 5% nonfat-dried milk (Sigma) PBS-Triton and probed with anti-HA (Sigma, 1∶1000) or anti-GAPDH (Covance, 1∶1000) antibodies and secondary HRP-conjugated antibody (1∶5000, Invitrogen). Proteins were visualized using ECL reagent according to the manufacturer’s instructions (Amersham Biosciences) using an ImageQuant LAS4000 imaging system and ImageQuant software (GE Healthcare, UK).

### Immunocytochemistry

Cells cultured on coverslips were washed, fixed in 4% PFA for 10 minutes, and permeabilised in 0.4% Triton in PBS for 10 minutes. Coverslips were then blocked in 5% milk-PBS for 30 minutes and anti-HA (Sigma, 1∶1000) or NF-200 (Sigma, 1∶200) was incubated overnight at 4°C. Cells were then washed and incubated in secondary fluorescent-conjugated antibody (1∶1000) (Molecular probes, Invitrogen) for 2 hours and mounted in DAPI-containing Vectorshield mounting media (Vectors lab). Axons, visualized by NF-200 staining, were measured using ImageJ software (NIH). A minimum of 15 cortical neurons was quantified. TUNEL staining for apoptotic cells was carried out as per the manufacturer’s protocol (Roche, UK). Confocal LSM510 (Zeiss) or fluorescence (Leica) microscopes were used to acquire images using Axiovision software (Zeiss).

### Organotypic Slice Culture

Organotypic slice cultures were performed from electroporated E14 whole embryos as previously described [27], [28]. Briefly, GFP containing vectors (1 µg/µl) with 0.5% Fast Green (Sigma) were injected using a microinjector (Picospritzer) into the lateral ventricle of the whole embryos. Success of DNA injection was determined by the visualization of Fast Green in the ventricular system. Electroporation was then performed on the whole head (skin and skull intact) with gold-coated electrodes (3×5mm GenePaddles, Havard Apparatus) using an electroporator setting (4×100 ms pulses separated by 100 ms long intervals at 55V) (ECM 830 Square Wave Electroporation system, Harvard Apparatus).

Brains were subsequently dissected from the embryos and placed in a dish of ice cold complete HBSS (HBSS 1x, 2.5 mM Hepes, 0.3 mM D-Glucose, 1 mM CaCl_2,_ 1 mM MgSo_4_, 4 mM NaHCO_3_ (Sigma/Gibco)), embedded in low melting point agar (Sigma) and sectioned at 250 µm and transferred to Poly-L-Lysine and laminin (Sigma)-pre-coated membrane inserts (BD Biosciences). Brain slices were cultured in slice culture medium (Basal Medium Eagle 0.7x, Complete HBSS 0.25x, 2.7 mM D-glucose, 0.1 mM L-glutamine and pen/strep (Gibco)) for 6 days. The cultured 250 µm slices were fixed in 4% PFA for 12–24 hours and subsequently embedded in low melt agarose gel (Sigma), re-sectioned at 30 µm thickness using a Vibratome, and slide mounted and imaged using a fluorescent microscope (Leica). Images were captured using Axiovision software (Zeiss).

### FACS sorting

Cortical cells electroporated on the day of dissociation and cultured for 4 days were washed with PBS and trypsinised (Gibco), scraped off plates and spun at 400×g for 5 minutes and washed twice in 1% BSA (Sigma) in PBS. They were resuspended in 0.5 ml of 1% BSA/PBS solution and GFP positive (GFP^+^) cells were immediately sorted for size and GFP signal using FACS on a Beckman Coulter MoFlo XPF machine and were subsequently used for RNA purification, microarray analyses and chromatin immunoprecipitation (ChIP) (see below).

### Transcriptional profiling

RNA was extracted from FACS sorted electroporated cortical cells using the RNeasy kit (Qiagen). The quality of the extracted RNA was assessed on a 2100 BioAnalyser using the RNA 6000 Pico Assay (Agilent Technologies). The RNA Integrity numbers were >7 for all samples. Fifteen nanograms of RNA from each of the 4 replicates were reverse transcribed, and the cDNA was amplified using the Ovation Pico system (NuGEN) according to manufacturer’s instruction. Two ‘no template’ controls were processed together with the samples, and no significant amplification due to contamination was found. Amplified double-stranded cDNA was transformed into single-stranded sense cDNA using the Ovation Exon Module (NuGEN). Sense cDNA was fragmented and labeled with biotin using the FL-Ovation cDNA Biotin Module V2 (NuGEN). Successful fragmentation was confirmed for all samples on the BioAnalyzer showing a peak sequence length of around 200 nt. Fragmented and labeled single-stranded sense cDNA was hybridized to Affymetrix Mouse Gene 1.0 ST arrays at 45°C overnight. Arrays were washed and then stained using the GeneChip Hybridization, Wash, and Stain Kit (Affymetrix) according to manufacturer’s instruction. The microarrays were scanned on an Affymetric GeneChip Scanner 3000. Data were normalized in GeneSpring (Agilent Technologies) using robust multi-array average (RMA) and PLIER analysis and differentially expressed genes were identified using the Welch *t*-test with a *p*-value cutoff of ≤0.05 and fold-change cut off of 1.5.

### Quantitative real-time RT-PCR (qRT-PCR)

Total RNA was purified from primary cortical cells using the RNeasy mini kit (Qiagen) and reversed transcribed using Sensiscript reverse transcriptase (Qiagen). Total cDNA was subject to real-time PCR on an ABI PRISM 7000 sequence detection system using SYBR green PCR master mix (Applied Biosystems) and primers are shown in Table S1. The housekeeping gene *Gapdh* was used as the internal normalising control. *Gapdh* has been used as a normalising control because its levels were not changed across all conditions tested as seen on our microarray data.

### Chromatin immunoprecipitation

The ChIP-IT enzymatic kit (Active Motif) was used per the manufacturer’s instructions. Briefly, cortical cells electroporated with either HA-IRES-GFP or Laf4-HA-IRES-GFP and cultured for 4 days were fixed with 1% formaldehyde for 20 minutes to cross-link DNA-protein interactions. After cross-linking was stopped with glycine, ∼4×10^6^ cells were washed with PBS and scraped in FACS buffer and cells were FACS sorted based on size and GFP signal as described above. Cross-linked chromatin from ∼1–2×10^5^ GFP^+^-cells was then enzymatically digested to yield 150–200 bp fragments and incubated overnight with magnetic beads plus 3 µg HA antibody (Sigma) or water at 4°C. This was followed by cross-linking reversal, proteinase K digestion, DNA extraction (PCR purification kit; Qiagen), and quantitative PCR (qPCR) with the primer sets listed in Table S1. The specific binding of HA-tagged Laf4 to promoters of *Mdga1* and *Mdga2* was calculated by subtracting the value of the ‘no antibody’ control and then normalized to the diluted input (1∶10). The percentage input was then calculated.

### Statistical Analysis

Prism GraphPad software was used for 1-way and 2-way ANOVA, with Bonferroni’s multiple comparison test when appropriate. Data are presented as mean ± SEM (*p<0.05; **p<0.01; ***p<0.001).

## Results

### *Laf4* expression during embryonic cortical development in the mouse

**Figure 1:**
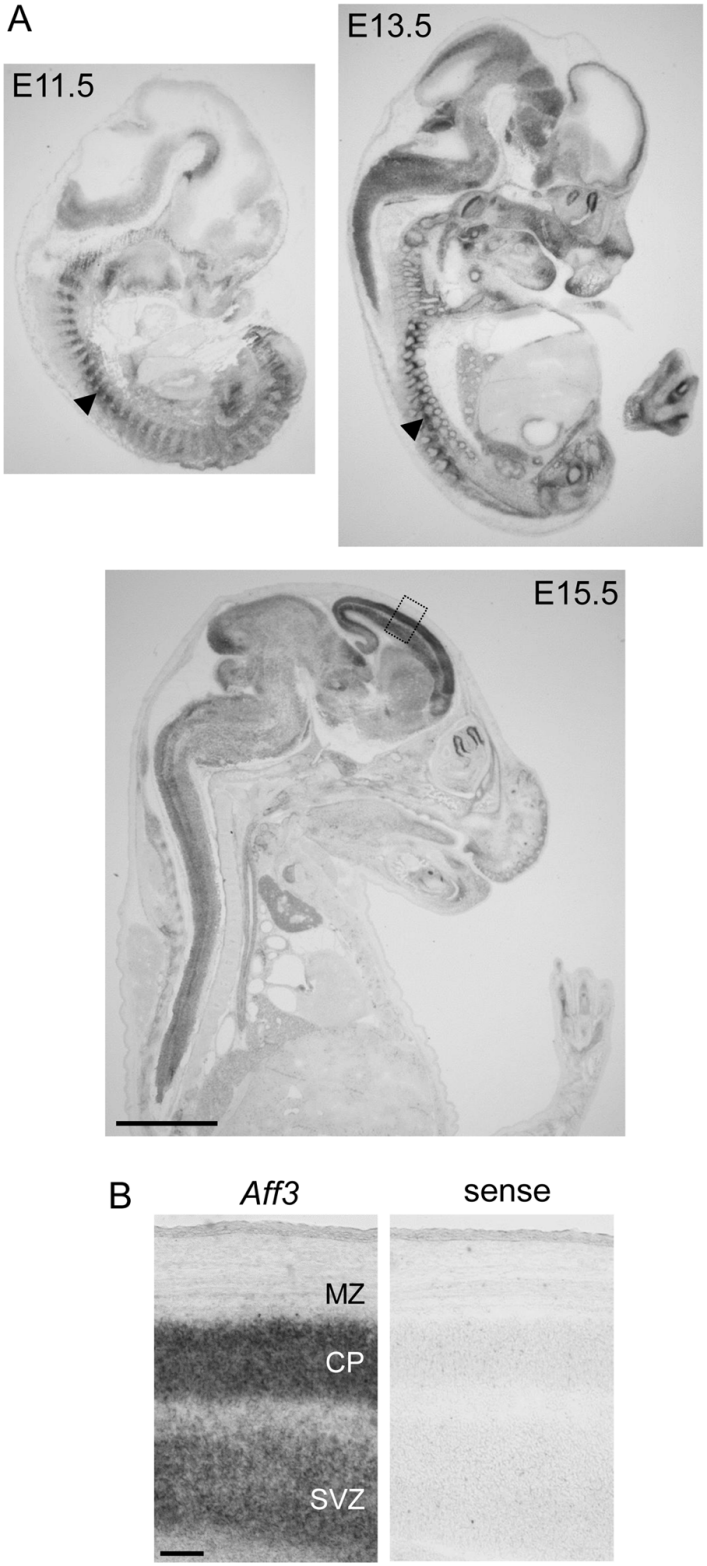
*Laf4* is expressed in the developing mouse cortex. *In situ* hybridisation of *Laf4* in the mouse embryo at E11.5, E13.5 and E15.5 (A). Expression is observed in the brain and somites (arrowheads) at E11.5 and 13.5 (A). By E15.5 expression is observed in the developing cortex below the medial zone (MZ) in the cortical plate (CP) and subventricular zone (SVZ) (B). The boxed region in (A) is shown in (B) with a control sense riboprobe from an adjacent section. Scale bars = 2 mm in (A), 100 µM in (B).

Previous studies in the mouse have demonstrated that *Laf4* expression is developmentally regulated [24]. However, to obtain a more comprehensive picture of the temporal pattern of *Laf4* in the embryonic cerebral cortex, a time course of expression analysis was carried out by *in* *situ* hybridization using a riboprobe designed to span all major *Laf4* transcripts. These data show that *Laf4* is detectable in the developing brain and somites at E11.5 as previously described [6] (**Figure 1A**). By E13.5, *Laf4* is highly expressed in the developing brain, and as embryogenesis proceeds, expression becomes localized to the subventricular zone (SVZ) and the dorsal cortex by E15.5 (**Figures 1A and B**); importantly, this corresponds to a developmental window during which intermediate progenitor cells in the SVZ proliferate and then subsequently migrate to form the upper layers of the cortex [29]. These data suggest that *Laf4* may be important for the maintenance and development of the embryonic cortex, in particular at key stages of neuronal migration and proliferation that are required to generate the correct patterns of cortical layering.

### Manipulation of Laf4 expression during cortical development

**Figure 2:**
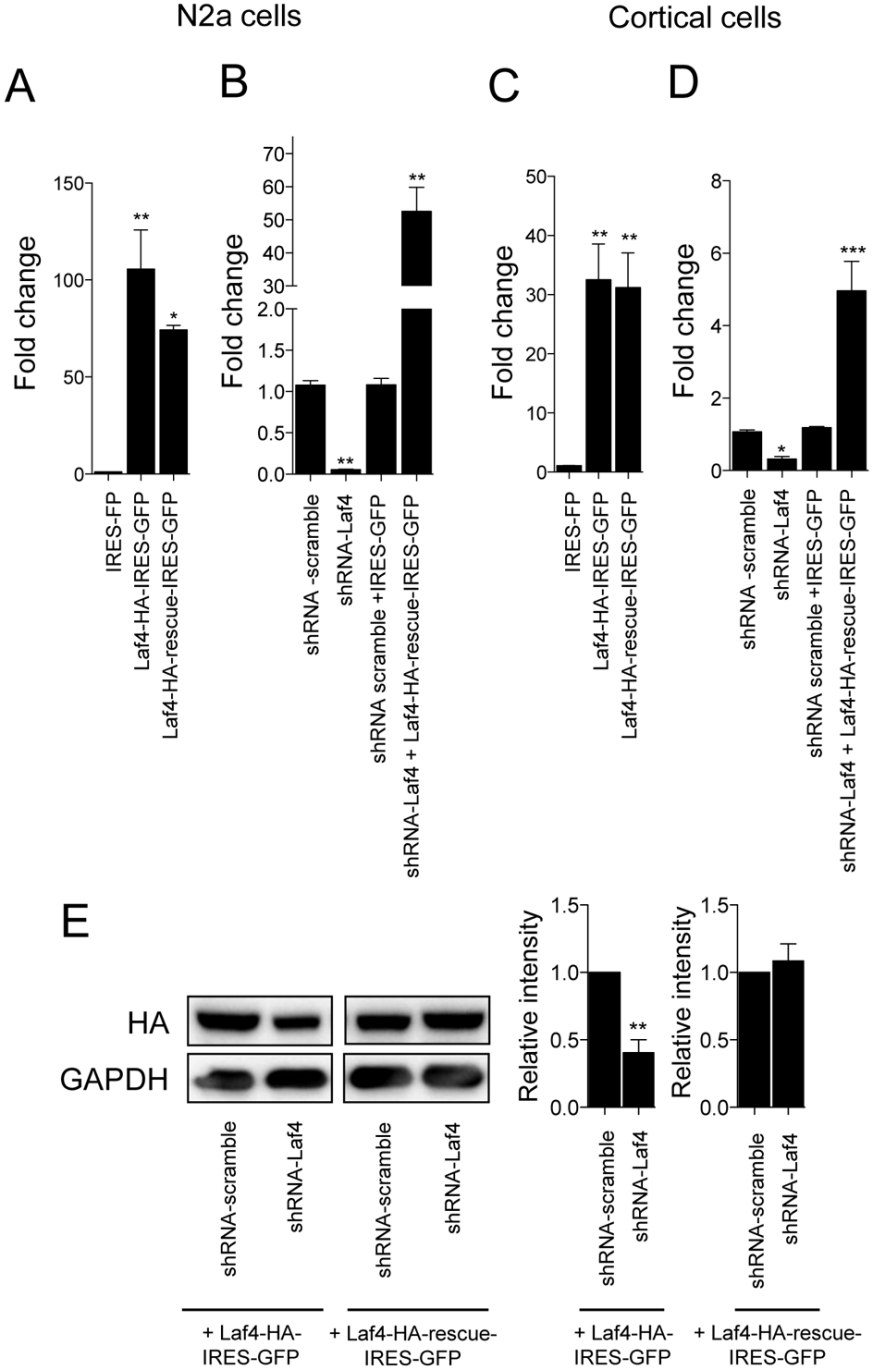
Knockdown of *Laf4* by shRNA. Quantitative RT-PCR of *Laf4* in N2a (A and B) and primary cortical cells (C and D). Comparative over-expression of HA-tagged Laf4 (Laf4-HA-IRES-GFP) and the shRNA control (Laf4-HA-rescue-IRES-GFP) occurs at similar levels in both cell types; data are shown versus endogenous *Laf4* (negative control HA-IRES-GFP vector) (A and C). Knockdown of endogenous *Laf4* in both cell types after transfection (B) or electroporation (D) with shRNA-Laf4 shows the recovery of *Laf4* expression using Laf4-HA-rescue-IRES-GFP. (E) Protein levels from over-expression of Laf4 (Laf4-HA-IRES-GFP), but not the control (Laf4-HA-rescue-IRES-GFP) construct is significantly knocked-down by the same shRNA*-*Laf4 in HeLa cells. The negative control shRNA (shRNA-scramble) has no effect on the levels of either endogenous *Laf4* (B and D) or exogenous over-expressed Laf4 (E). In all panels, quantification of three independent repeats in shown (*p<0.05, **p<0.01, ***p<0.001, 1-way ANOVA Bonferroni’s multiple comparison test).

In order to investigate the potential role of *Laf4* in cortical migration, *Laf4* expression was manipulated in mouse embryonic brains. Prior to these studies, the selected shRNA construct (shRNA-Laf4) was tested in both N2a and primary cortical cells resulting in a ∼95% or ∼80% knockdown of endogenous *Laf4* levels respectively, as quantified by qRT-PCR (**Figures 2B and D**). Importantly, a scramble control shRNA (shRNA-scramble) had no effect on endogenous *Laf4* levels (**Figures 2B and D**). The knockdown efficiency of the same shRNA-Laf4 construct was further assessed, demonstrating that even high levels of exogenous Laf4 protein expression (by co-transfection of a Laf4**-**HA**-**IRES**-**GFP construct) could be significantly ablated at the protein level (**Figure 2E**). The specificity of knockdown was also confirmed by co-transfection with a construct that contained a mutated version of Laf4 that would not be recognized by the shRNA (Laf4-rescue-HA-IRES-GFP; **Figures 2A–E**).

**Figure 3:**
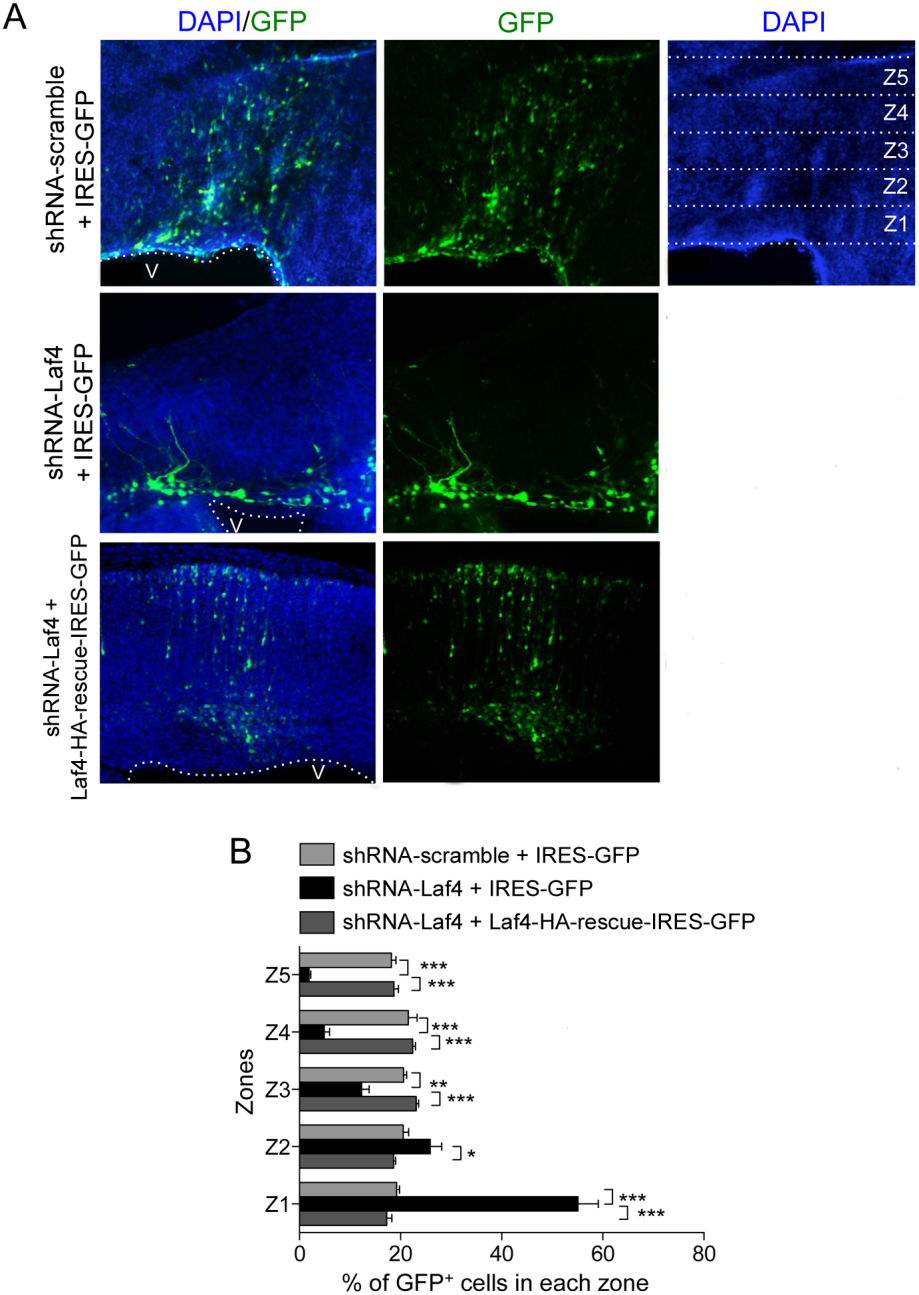
Knockdown of *Laf4* causes a defect in the migration of cortical cells. E14 mouse brains were electroporated with shRNA-Laf4 or shRNA-scramble vectors with a control (IRES-GFP) or the Laf4-rescue-IRES-GFP vector and cultured for six days before re-sectioning for image analysis (A). The average percentage of GFP^+^ cells in 5 zones (example zones Z1–Z5 shown) spanning from the ventricle (V) to the pial border was quantified (B). Brains electroporated with shRNA-Laf4 construct show a significant reduction in GFP^+^ cells corresponding to migrating cells to the superficial layers of the cortex; this phenotype is almost completely reversed by co-electroporation with a Laf4-rescue-IRES-GFP construct. (*n* = 6 brains quantified for each vector combination; *p<0.05, **p<0.01 (2-way ANOVA Bonferroni’s multiple comparison test). Scale bar = 50 µM.

To then study cortical migration, shRNA and control (IRES-GFP) plasmids were injected *ex utero* into the lateral ventricle of E14.5 whole mouse embryos followed by electroporation and organotypic slice culture from dissected brains. After six days the cortical slices were re-sectioned and the number of GFP^+^ neurons was counted; the image of each section was divided equally into five bins spanning the cortex using the ventricular and pial borders as anatomical landmarks as previously described (**Figure 3A**) [30]. Embryos electroporated with a negative control scramble shRNA showed a robust migration of GFP^+^ cells to the cortical pial border as expected (**Figure 3A**). However, a reproducible reduction in migration was observed when the knockdown shRNA-Laf4 construct was expressed (**Figure 3A**); 80% of the cells from knockdown cultures were located in bins 1 and 2 beneath the developing cortical plate, as opposed to a relatively even distribution amongst the layers when the scramble shRNA was electroporated (**Figure 3B**). Importantly, cortical slice cultures co-electroporated with shRNA-Laf4 and Laf4-rescue-HA-IRES-GFP did not show any migration phenotype (**Figures 3A and B**), suggesting that a specific cortical migration deficit is the result of reducing *Laf4* expression during early development.

**Figure 4:**
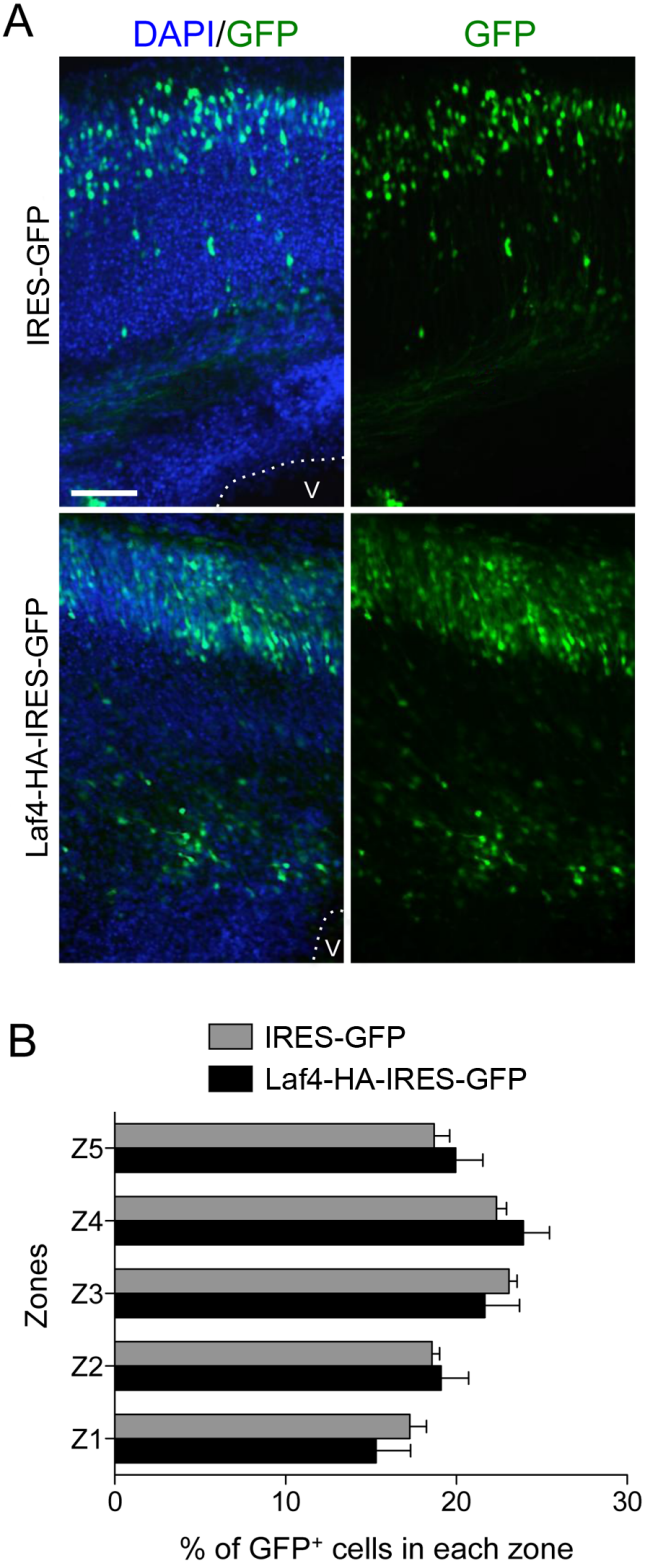
Overexpression of Laf4 does not affect significantly migration of cortical cells. E14 mouse brains were electroporated with Laf4-HA-IRES-GFP or control (HA-IRES-GFP) vectors and cultured for six days before re-sectioning for image analysis (A). The average percentage of GFP^+^ cells in 5 zones spanning from the ventricle (V) to the pial border was quantified (B) and no significant difference (*p* = 1.00, 0.27, 0.71, 0.80, 0.63 for Z1 to Z5 respectively) was observed between Laf4-HA-IRES-GFP or the control vector (*n* = 6 brains quantified for each vector, 2-way ANOVA Bonferroni’s multiple comparison test). Scale bar = 50 µM.

To assess the significance of increasing Laf4 expression at the same point in development, slice cultures were analysed as above from embryos electroporated with the Laf4-HA-IRES-GFP or IRES-GFP constructs (**Figure 4A**). Quantification showed that there was no significant difference in cortical migration (**Figure 4B**), suggesting that the presence of exogenously-expressed Laf4 is not detrimental to cortical development. To further assess the consequences of manipulating *Laf4* levels, transfection of over-expression, knockdown and control constructs was carried out in primary cortical culture, showing no alteration in cell survival or axon growth between conditions (**Figure S1**).

### Transcriptional analysis of Laf4 over-expression

Since members of the AFF family are known to act as transcriptional modulators and Laf4 appeared to be influencing cortical development, primary cortical neurons were screened for potential Laf4 target genes related to the observed migration phenotype by expression profiling. It has been shown previously that over-expression of AFF proteins can be used to identify both downstream and direct targets of the AFF transcriptional complex [22]. Therefore, over-expression of *Laf4* using the Laf4-HA-IRES-GFP construct was carried out in primary cortical cells and compared with cells expressing the control IRES-GFP vector. To minimize potential non-specific effects of non-transfected cells, FACS sorting was used to select transfected cells on both the basis of size and GFP expression. Transcriptional profiling using Affymetrix microarrays identified a number of genes that were differentially expressed between replicates of *Laf4* over-expressing and control cell populations (**Table 1**). Interestingly, of the transcripts deregulated by more than 1.5-fold, over three-quarters were up-regulated; the most highly over-expressed of these was *Aff3/Laf4*.

**Table 1:** Differentially expressed transcripts from Laf4 over-expression in primary cortical cells.

Gene name	Fold change	P-value	Direction
*Aff3*	2.5	1.00E-04	up
*Gatad1*	2.0	0.00876146	up
*Ndufs5*	2.0	0.01630139	up
*Snord35a*	1.8	0.02089867	up
*Zfp780b*	1.8	0.03654183	up
*Txlng*	1.8	0.00685382	up
*Txlng*	1.8	0.01637254	up
*Scn3a*	1.7	0.01588485	up
*Lemd3*	1.7	0.03688544	up
*Rrm2b*	1.7	0.01885046	up
*Lphn3*	1.7	0.03866969	up
*Nop56*	1.7	0.01139775	up
*Snord33*	1.7	0.0306814	up
*Ptdss1*	1.6	0.00358072	up
*Rfesd*	1.6	0.02712189	up
*Hist1h2bc*	1.6	0.04939594	up
*Nop16*	1.6	0.02797953	up
*Trpm7*	1.6	0.01894537	up
*A230046K03Rik*	1.6	0.02141088	up
*Braf*	1.6	0.01453028	up
*Samm50*	1.6	0.02693954	up
*1110012L19Rik*	1.6	0.0234425	up
*Kdm5b*	1.6	0.01495683	up
*Ercc3*	1.6	0.00651398	up
*Mga*	1.6	0.00228502	up
*Mdga2*	1.5	0.03499631	up
*Dcun1d4*	1.5	0.04789757	up
*Btaf1*	1.5	0.00433972	up
*Gpr137b-ps*	1.5	0.01491051	up
*Mastl*	1.5	0.00317947	up
*4932438A13Rik*	1.5	0.01460414	down
*Olfr1174-ps*	1.5	0.02718545	up
*Vps72*	1.5	0.02154237	up
*Gm9958*	1.5	0.01058675	down
*Gm20091*	1.5	0.00420859	up
*Rbbp8*	1.5	0.01891017	up
*Gbe1*	1.5	0.03423288	up
*Trps1*	1.5	0.01591994	up
*2210408I21Rik*	1.5	0.02667212	up
*Jhdm1d*	1.5	0.02668253	up
*Dpy19l4*	1.5	0.01150993	up

**Figure 5:**
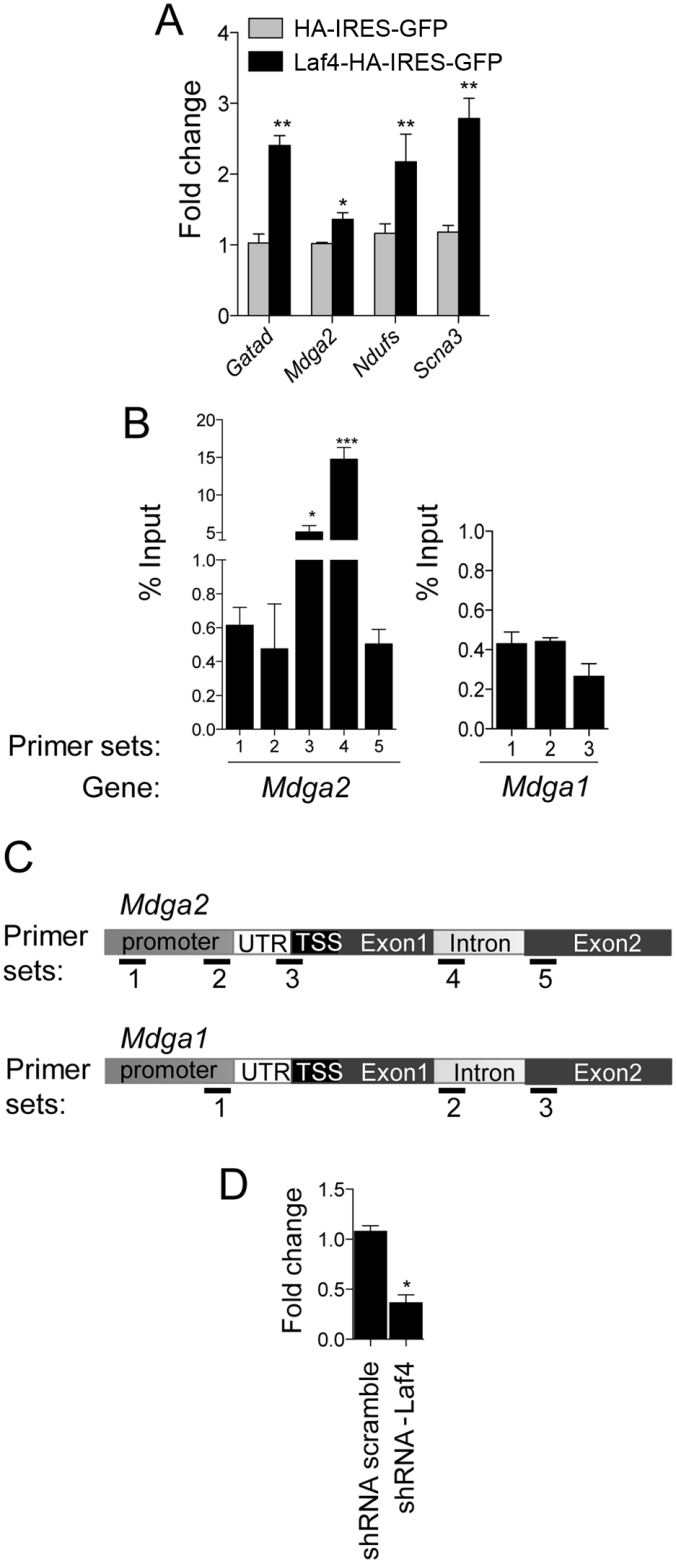
Identification of Laf4 as a potential transcriptional regulator of *Mdga2*. (A) qRT-PCR analysis of four genes originally shown to be up-regulated from microarray transcriptome profiling of FACS-sorted primary cortical cells over-expressing Laf4 (Laf4-HA-IRES-GFP) compared to an empty control (HA-IRES-GFP) vector. Significant up-regulation of *Gatad1, Mdga2, Ndufs* and *Scna3* was confirmed in *Laf4* over-expressing cells (*n* = 3 biological replicates *p<0.05, **p<0.01, 2-way ANOVA Bonferroni’s multiple comparison test). (B) ChIP from cortical cells transfected and FACS-sorted as in (A) was performed using an anti-HA antibody to HA-tagged Laf4-IRES-GFP or empty vector (HA-IRES-GFP). qPCR showed specific binding of Laf4 to *Mdga2* at the beginning of the coding region (primer pair 3 and 4 shown in (C)) and at the end of the first exon/beginning of the first intron (primer pair 4). Laf4 did not appear to bind to *Mdga1* for the primers tested. (*n* = 3 biological replicates; *p<0.05, ***p<0.001). 1-way ANOVA Bonferroni’s multiple comparison test used to compare each primer sets to primer 5 (Mdga2) or 3 (Mdga1) used as control primer set. (D) qRT-PCR analysis of *Mdga2* levels in cortical cells transfected with shRNA-Laf4 vector (*p<0.01, 1-way ANOVA Bonferroni’s multiple comparison test).

In order to validate the microarray results, a number of genes were selected for qRT-PCR analysis. Genes were prioritized based on known expression in the brain and in particular overlapping spatial-temporal expression with *Laf4*. Four genes were selected for further validation: *Gatad1, Ndufs5, Mdga2* and *Scn3a*; independent samples of FACS sorted primary cortical cells were used for these experiments. In agreement with the microarray profiling, all four targets showed up-regulation when *Laf4* was over-expressed compared to cells transfected with the control vector (**Figure 5A**).

### *Mdga2* is a potential direct target of Laf4

Of the confirmed microarray targets, *Mdga2* (MAM domain containing glycosylphosphatidylinositol anchor 2) is a cell adhesion molecule from an immunoglobulin superfamily that is particularly relevant to cortical development. *Mdga2* is highly expressed in the developing rodent brain and has been highlighted as an important factor for normal outgrowth of commissural interneurons in the chick [31]. To determine whether Laf4 or a Laf4-containing complex is able to bind in or around the *Mdga2* locus as a potential direct transcriptional regulator [22], ChIP combined with qPCR analysis was performed in primary cortical cells electroporated with either HA-IRES-GFP or Laf4-HA-IRES-GFP. The results indicate that Laf4 binds to the *Mdga2* locus, particularly towards the transcription-start site (TSS) and the end of exon 1 (**Figures 5B and C**); a significant increase in Laf4 occupancy was observed from primary cortical cells electroporated with Laf4-HA-IRES-GFP versus a control HA-IRES-GFP construct. We also tested the binding of Laf4 on the *Mdga1* promoter, a gene closely related to *Mdga2*. However, for the primers tested, no binding of Laf4 was detected suggesting that Laf4 might bind specifically to the *Mdga2* promoter (**Figure 5B–C**). These data suggest that a Laf4-containing transcriptional complex is able to specifically bind to the *Mdga2* locus to regulate its expression. To further analyse the transcriptional relationship between Laf4 and *Mdga2*, we carried out a reciprocal experiment whereby five independent shRNAs specific to either Laf4 3′-UTR (shRNA Laf4 #08) or various exons (shRNA Laf4 #09 to #12) were tested in N2a cells. In line with the results above, a 50–70% knockdown of endogenous *Laf4* resulted in a corresponding 50–60% reduction in *Mdga2* expression (**Figure S2**). In addition, using the shRNA used for the migration assays (shRNA-Laf4) in N2a cells, a 74.5% reduction in *Mdga2* expression results from 95.2% knockdown of *Laf4* (**Figure S2**). These data suggest that even the predicted 50% loss of *LAF4* expression in humans, either from heterozygous deletion or CCG repeat silencing, is sufficient to influence the transcription of Laf4 target genes.

### Over-expression of *Mdga2* rescues the *Laf4* knockdown-related migration phenotype

**Figure 6:**
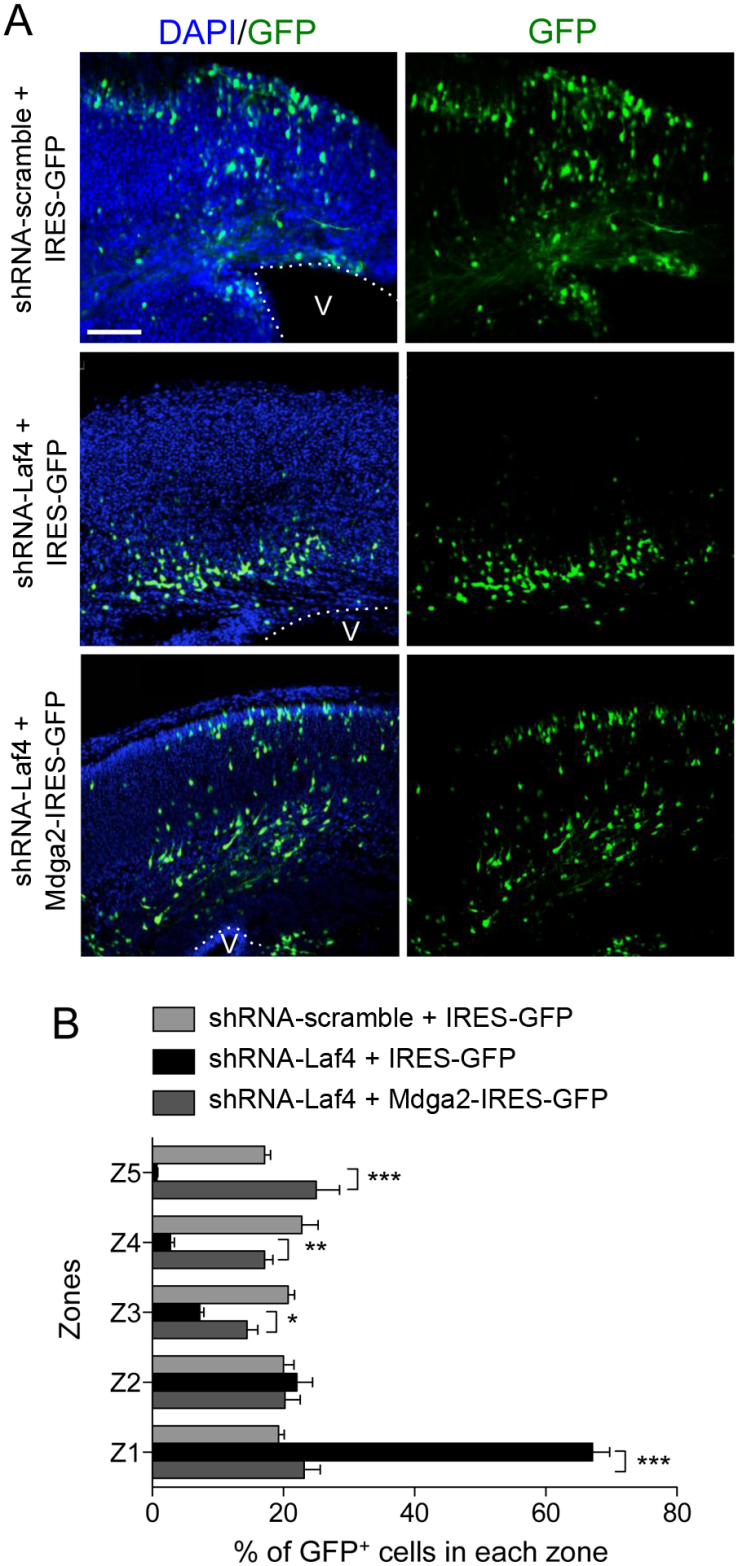
Over-expression of *Mdga2* rescues the deficit of cortical cells migration generated by *Laf4* knockdown. (A) E14 mouse brains were electroporated with shRNA-Laf4 or shRNA-scramble vectors with the control IRES-GFP or an Mdga2-IRES-GFP vector and cultured for six days before re-sectioning for image analysis. The average percentage of GFP^+^ cells in 5 zones spanning from the ventricle (V) to the pial border was quantified (B). Co-electroporation of shRNA-Laf4 and Mdga2-IRES-GFP constructs results in rescue of the migration defect observed using shRNA-Laf4 alone. (*n* = 4 brains quantified for each vector combination; *p<0.05, **p<0.01, ***p<0.001, 2-way ANOVA Bonferroni’s multiple comparison test).

As *Mdga2* is known to play an important role in neuronal outgrowth and may be regulated by Laf4, it was investigated whether increasing the levels of *Mdga2* might influence the migration phenotype observed using *Laf4* knockdown. Using the same method as above (**Figure 3**), cortical slice cultures were co-electroporated with shRNA-Laf4 and Mdga2-IRES-GFP (**Figure 6A–B**). Importantly, these cultures displayed a phenotype almost identical to normal control slices, whereas those electroporated with shRNA-Laf4 alone still showed the stalled migration pattern as observed previously (**Figures 6A** **and** **3A**). This suggests that over-expression of *Mdga2* can alleviate the neuronal migration deficit caused by *Laf4* knockdown. Therefore not only do these data support a role for Mdga2 in migration, but also provide evidence that regulation of *Mdga2* by Laf4 may be important in this process.

## Discussion

### Laf4 is the first AFF member identified as a regulator of cerebral cortex development

Although *AF4*, *AF5Q31* and *LAF4* are highly expressed in human embryonic brains [8], [25], a functional role for these genes during neurodevelopment has not been described. We showed here that *Laf4* is expressed as early as E13.5 in the mouse neocortex and demonstrated a direct function for this gene in migration of cortical neurons. It is noteworthy that the AFF genes *Fmr2* and *Af4* are also expressed in the mouse cortical plate, but later in development than *Laf4* [32]–[34], suggesting that *Laf4* may have a specific and unique role at the earlier embryonic time-points studied here. Given the degree of sequence conservation within the ALF family [35], our present study potentially has implications for AFF-associated disorders of the CNS such as FRAXE mental retardation associated with FMR2 [36]–[38]. Interestingly, *Fmr2* knockout mice show a very mild phenotype, displaying memory impairment and abnormalities in nociception [11]. While no cortical migration deficits have been noted in these mice, no detailed anatomical analysis was described [11]. However, more recently Fmr2 has been shown to regulate the transcription of *Jun*, a gene previously implicated in neuronal migration [23], [39]. Therefore in light of these studies and our data, in addition to LAF4 mutations associated with cortical atrophy and ID, there is increasing evidence that AFF proteins play important roles in neurodevelopment.

AFF proteins were originally described as putative transcription factors based on the presence of a conserved transactivation domain [16]; however, only very few transcriptional targets have been confirmed to date [22]. Our study suggests that *Mdga2* gene is under the transcriptional control of Laf4. Interestingly, of the genes deregulated in primary cortical cells by more than 1.5-fold from Laf4 over-expression, 84% were up-regulated. This suggests that Laf4 has a stimulatory role on gene transcription, consistent with data obtained from the Af4-containing complex [17], [40]. This is also in accordance with a previous study showing that LAF4, like AF4 and AF5Q31, interacts with AF9/ENL protein, positive transcription elongation factor b (P-TEFb) and histone-H3 methyltransferase DOT1L [41]. In further support of Laf4 function as a transcription regulator, overexpression of *Laf4* in cortical cells led to an increase in BTAF1 RNA polymerase II (*Btaf1*), part of the pre-initiation transcription factor II D complex [42], and Max gene associated (*Mga*) which binds to the transcription factor Max, a facultative component of the MLL1 complex [43]. This suggests that Laf4 likely acts in collaboration with other transcription factors or chromatin remodellers to control gene transcription.

### Mdga2, a direct transcriptional target for Laf4, is important for cortical development

Here, we demonstrate for the first time that Mdga2 is involved in cell migration during the process of cortical layering; indeed *Mdga2* over-expression was able to rescue the migration deficit resulting from *Laf4* knockdown. It is noteworthy that while *Mdga2* over-expression largely rescued the deficit, there was not a complete rescue. Interestingly, *Mdga1*, *Mdga2* closest homologue, is required for neuronal migration [44], [45]. Thus, while Mdga2 plays a major role in Laf4-regulated migration, it may not be the only effector gene.

Recent studies have shown that reduced levels of *Mdga2* in open-book preparations from chicken embryos affect rostral growth of commissural axons [31]. Moreover, Mdga2, like Mdga1, binds to neuroligin-2 [46], [47], and binding of Mdga1 to neuroligin-2 inhibits neuroligin-2 synapse-promoting activity leading to reduction of synapse development and synaptic transmission in culture [46], [47]; importantly, neuroligin-2 is expressed in the mouse brain from E18 to P25 [47]. Together, these studies suggest that, in addition to its role in cellular migration, Mdga2 may also affect axon growth and synapse formation in the developing cortex.

### Importance of cell migration deregulation in disease

Our current study has shed some important new light on the potential mechanisms underlying the developmental delay and cortical atrophy observed from a heterozygous *LAF4* deletion in humans and repeat expansion silencing of the gene associated with ID [5], [6]. The cell migration data presented here may represent a greater degree of *LAF4* knockdown than observed in these human conditions; but importantly, testing of additional shRNAs demonstrated that knockdown of *Laf4* to levels that might be expected by haploinsufficiency results in alterations in expression of a transcriptional target of Laf4. Consequently, our results suggest that loss of *LAF4* expression may have resulted in defective cell migration during early cortical development. Mutations in genes involved in neuronal cell migration have previously been associated with developmental delay and ID in humans [6], [48], [49]. For example, doublecortin is associated with band heterotopia, a condition with severe intellectual disability and epilepsy, and which has been shown to be a consequence of radial migration deficits during neocortex development [50]. Doublecortin is also associated with lissencephaly, a disease presenting severe ID, seizures and brain with reduced gyration [48]. Similarly, mutations in *LIS1*, a gene involved in radial cortical neuronal migration, have also been associated with lissencephaly [49], [51], [52]. Moreover, in accordance with a role of *Mdga2* in cellular migration during brain development, previous large-scale human genetic analyses have associated variations in *MDGA* genes with schizophrenia, bipolar disorder and autism spectrum disorder [53]–[56]. Taken together, our study demonstrates the key transcriptional role of *Laf4* during cortical cell migration that is relevant to the haploinsufficiency and silencing associated with human neurodevelopmental disorders.

## Supporting Information

Figure S1 **Modulation of Laf4 levels in cortical cells does not affect survival or axon growth.** (A–C) Primary cortical cells were electroporated with the constructs indicated and cultured for 60 hours. (A) Cell death as assessed by TUNEL staining showed no significant difference in the number of apoptotic cells detected across all conditions. (B–C) Axons were visualised by NF-200 immunostaining (examples shown in (B)) and quantified, showing that axon length is not affected by levels of Laf4 or Mdga2. Scale bar: 50 µm.
(TIF)

Figure S2 **Levels of** ***Mdga2*** **are correlated with levels of** ***Laf4.*** (A–B) N2a cells were transfected for 48 hours with one of five additional shRNA constructs against *Laf4* (#8–#12) and levels of *Laf4* (A) and *Mdga2* (B) were quantified by qRT-PCR. Three out of the five (#11 and #12) additional shRNA constructs led to significant decrease of *Laf4* endogenous levels (A) and this was correlated with a significant decrease in *Mdga2* level, as also observed using the shRNA construct used for the slice culture experiments (shRNA-Laf4) (B). *p<0.05, 2-way ANOVA Bonferroni’s multiple comparison test.
(TIF)

Table S1 **Primers used in this study.**
(DOC)
