# Supplementary figures and images for: Laf4/Aff3, a Gene Involved in Intellectual Disability, Is Required for Cellular Migration in the Mouse Cerebral Cortex
Source: PLoS One. 2014 Aug 27;9(8):e105933. doi: 10.1371/journal.pone.0105933 (PMC4146563; doi:10.1371/journal.pone.0105933)

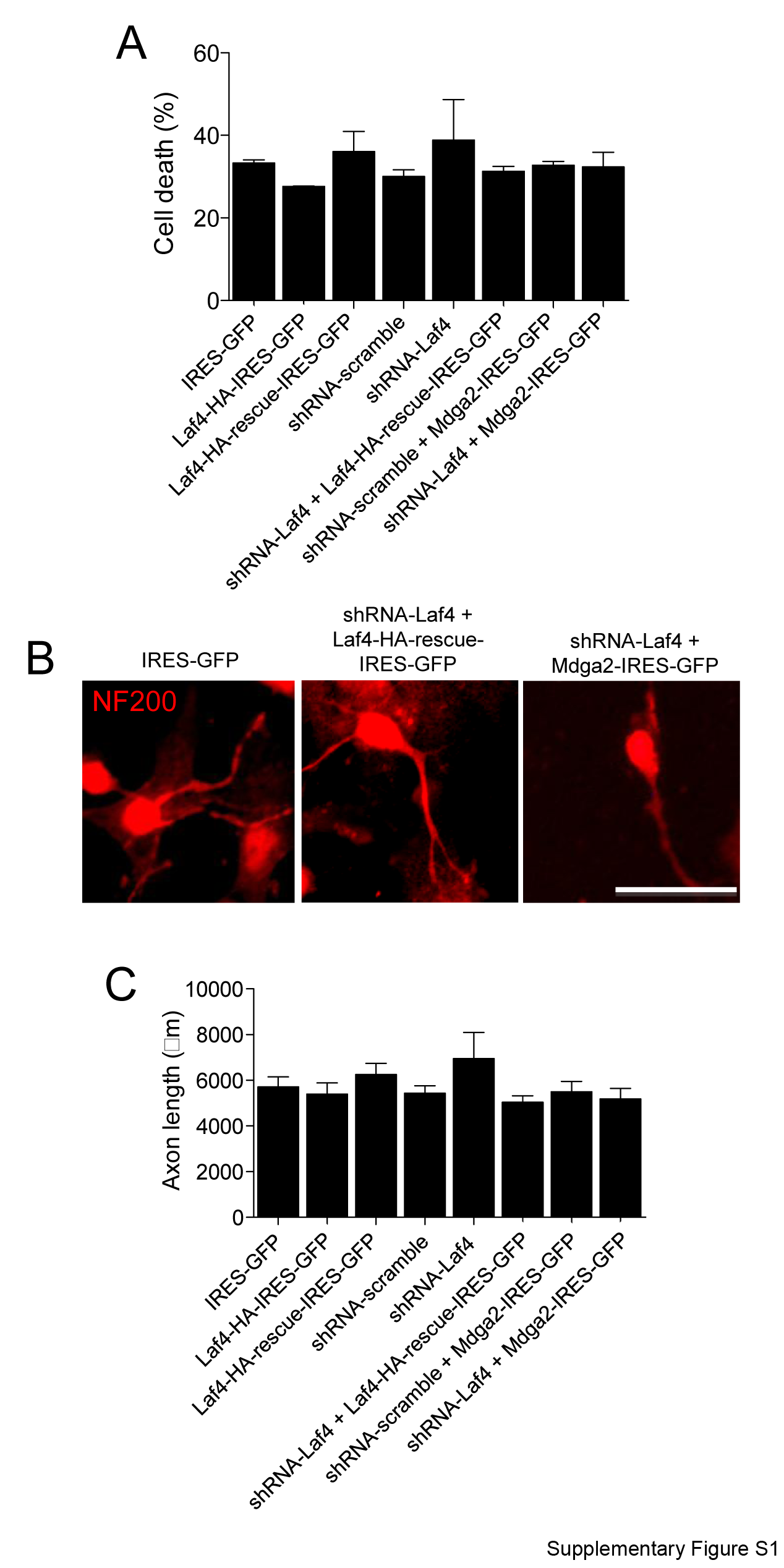

Supplement: Figure S1 — Modulation of Laf4 levels in cortical cells does not affect survival or axon growth. (A–C) Primary cortical cells were electroporated with the constructs indicated and cultured for 60 hours. (A) Cell death as assessed by TUNEL staining showed no significant difference in the number of apoptotic cells detected across all conditions. (B–C) Axons were visualised by NF-200 immunostaining (examples shown in (B)) and quantified, showing that axon length is not affected by levels of Laf4 or Mdga2. Scale bar: 50 µm. (TIF) [file pone.0105933.s001.tif]

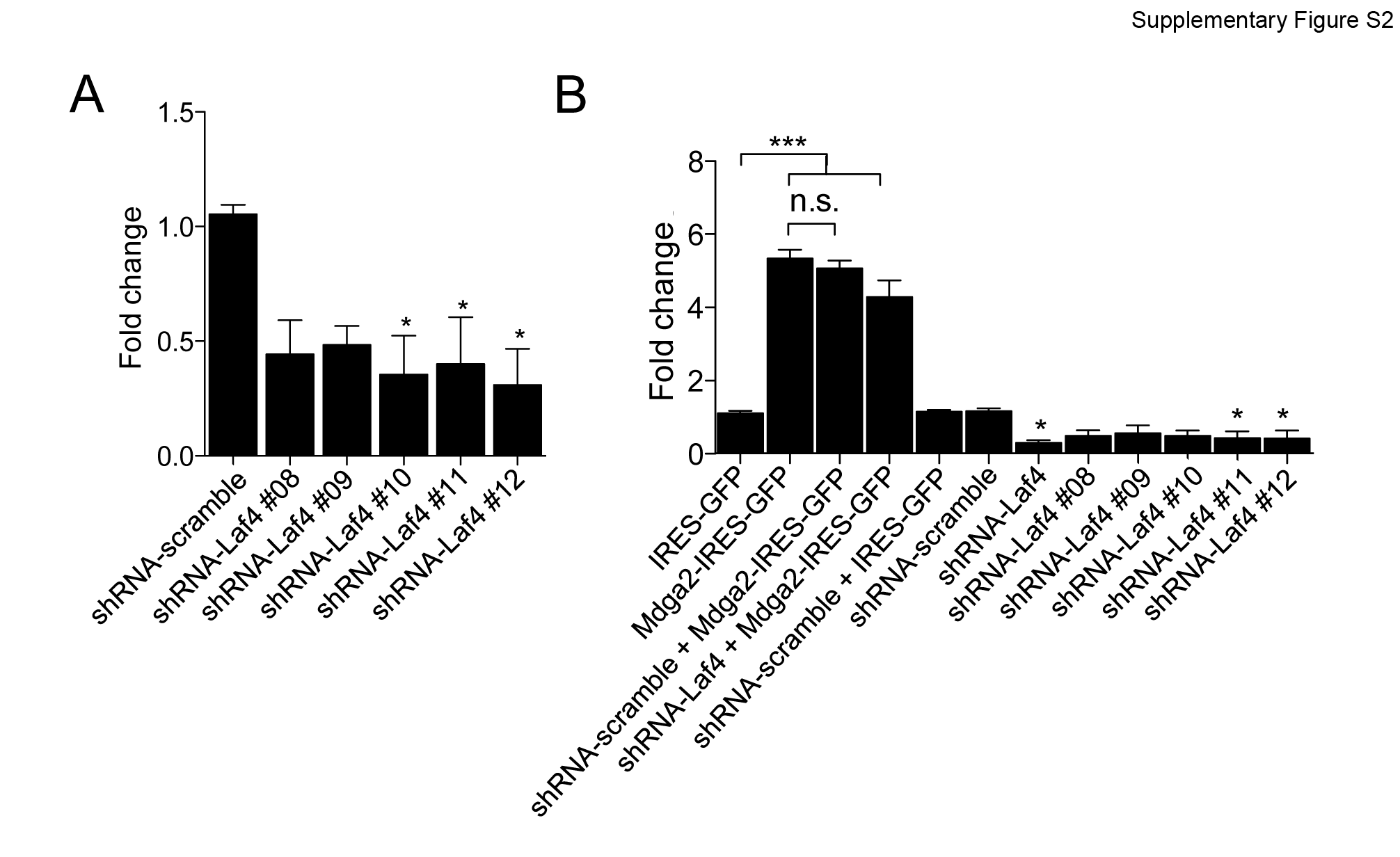

Supplement: Figure S2 — Levels of Mdga2 are correlated with levels of Laf4. (A–B) N2a cells were transfected for 48 hours with one of five additional shRNA constructs against Laf4 (#8–#12) and levels of Laf4 (A) and Mdga2 (B) were quantified by qRT-PCR. Three out of the five (#11 and #12) additional shRNA constructs led to significant decrease of Laf4 endogenous levels (A) and this was correlated with a significant decrease in Mdga2 level, as also observed using the shRNA construct used for the slice culture experiments (shRNA-Laf4) (B). *p<0.05, 2-way ANOVA Bonferroni’s multiple comparison test. (TIF) [file pone.0105933.s002.tif]
