# Supplementary material for: Laf4/Aff3, a Gene Involved in Intellectual Disability, Is Required for Cellular Migration in the Mouse Cerebral Cortex
Source: PLoS One. 2014 Aug 27;9(8):e105933. doi: 10.1371/journal.pone.0105933 (PMC4146563; doi:10.1371/journal.pone.0105933)
Supplement: Table S1 — Primers used in this study. (DOC) [file pone.0105933.s003.doc]

**Supplementary Table S1: Primers used in this study**

| **Name** | **Sequence** |
| --- | --- |
| **shRNAs** |  |
| shRNA-Laf4 F | TG GACCTTGCTTGGAGATGGTGG TTCAAGAGAACACCATCTCCAAGCAAGGTCCTTTTTC |
| shRNA-Laf4 R | TCGAGAAAAAGGACCTTGCTTGGAGATGGTGTTCTCTTGAACCACCATCTCCAAGCAAGGTCCA |
| shRNA-scramble F | TGCAACAAGATGAAGAGCACCAA TTCAAGAGATTGGTGCTCTTCATCTTGTTGCTTTTTC |
| shRNA-scramble R | TCGAGAAAAAGCAACAAGATGAAGAGCACCAATCTCTTGAATTGGTGCTCTTCATCTTGTTGCA |
| **ChIP primers** |  |
| Mdga2 1F | TCCTCCCTCTCCTGCAAGTG |
| Mdga2 1R | TGGAGGCAGCACCAATCTC |
| Mdga2 2F | CACAGCTCTGCTTCCCATCA |
| Mdga2 2R | ACAATACCCCAATACATGCACTCA |
| Mdga2 3F | CGCTGGCCTCCAGAAGGT |
| Mdga2 3R | CAGCCACACGAGACCGTACA |
| Mdga2 4F | CATCGCACTCAGAGGAACGA |
| Mdga2 4R | AAGACTGCAAGAGCCCATGTTT |
| Mdga2 5F | ACTCTAGAATTGACCTGCCTGGTT |
| Mdga2 5R | AGGCGCTTCCTGCTGTTTT |
| Mdga1 1F | TGTCCACTTGTCCTCCCTAAGC |
| Mdga1 1R | GCTAATGGAGTGAAGGCAACCT |
| Mdga1 2F | GATCCATCAAAATGCATAGAAATGA |
| Mdga1 2R | GAGGCAGCACTGGACTGACA |
| Mdga1 3F | GCCCGCTGCGCTTTG |
| Mdga1 3R | ACACGGTTTCACTGCAGCAA |
| **qPCR primers** |  |
| Gatad1 F | ACGTCGTCCTCCATGTGGAA |
| Gatad1 R | CGGCCCGTGCAGTGA |
| Ndufs5 F | CAGCGGGAGAAGCTAATGAAA |
| Ndufs5 R | TCCTCCCTGCCCGAATG |
| Scn3a F | TGCTGACGGTGGGAAACC |
| Scn3a R | TGCGATGATTTTCAGGACCAT |
| Mdga2 F | CTCGACTTCTCAGCCCTGTA |
| Mdga2 R | TTCCCACTCGACGACCATAG |
|  |  |
|  |  |
|  |  |
